# Development of an immunodeficient mouse that allows for conditional ablation of monocytic cells

**DOI:** 10.3389/fimmu.2025.1699385

**Published:** 2025-11-04

**Authors:** Alvin J. Hui, Kelly Lai, Eva H. Doyle, Guadalupe Rivera-Torruco, Alan G. Gutierrez, Emily J. Du, Yarah B. M. Meijer, Gabrielle Smith, Renata Gilfanova, Kenton G. Chung, Kirsten M. Auclair, Pamela Milani, Rachael P. Jackman, Johnson Q. Tran, Marcus O. Muench

**Affiliations:** ^1^ Vitalant Research Institute, San Francisco, CA, United States; ^2^ Department of Laboratory Medicine, University of California, San Francisco, San Francisco, CA, United States

**Keywords:** macrophage - cell, phagocyte, hemolytic complement, cell chimeras, humanized mice, xenotransplantation, blood cell transfusion, myeloid cells

## Abstract

**Background and aims:**

Immunodeficient mice, like the NOD-SCID-Gamma (NSG) strain, are important for the study of xenogeneic cells because of their lack of lymphocytes, dysfunctional hemolytic complement factor 5 (C5), and macrophage defects making them permissive hosts. Nonetheless, cellular barriers remain that limit engraftment of foreign cells such as monocytic phagocytes. Accordingly, we created a line of mice that allows for depletion of monocytic cells by breeding NSG mice with macrophage Fas-induced apoptosis (MaFIA) mice resulting in a stable line of NSG-MaFIA mice.

**Methods:**

NSG-MaFIA mice were generated by crossing NSG and MaFIA mice, with the hybrids backcrossed for nine generations to NSG mice. Flow cytometry was used to detect the expression of the MaFIA gene construct among blood leukocytes. Functional and confirmatory studies evaluated the successful transfer of the MaFIA transgene into the NSG genetic background. Apoptosis of monocytic cells was achieved through administration of a homodimerizer drug. The phenotypic characteristics of NSG mice were confirmed in NSG-MaFIA mice by flow cytometry, CBC analysis, testing of radiation sensitivity, and sequencing of the C5 gene. The permissiveness of NSG-MaFIA mice for xenogeneic engraftment was tested by transfusion of human red blood cells (RBCs) and peripheral blood mononuclear cells (PBMCs).

**Results:**

The MaFIA transgene was hybridized into NSG mice as exhibited by expression of a fluorescent marker. Functional expression of the MaFIA transgene was evidenced by weight loss and decreased fluorescence after homodimerizer treatment. NSG-MaFIA mice are lymphopenic, are sensitive to X-ray irradiation, and carry a mutated C5 gene. Transfusion of human RBCs resulted in similar clearance in NSG and NSG-MaFIA mice, without homodimerizer treatment, indicating a similar innate immune response. Moreover, transfusion of human RBCs or PBMCs after depletion of monocytic cells led to prolonged circulation of RBCs and rapid engraftment of leukocytes.

**Conclusions:**

A novel NSG-MaFIA mouse line was developed that has use in the study of monocytic cells and in the development of better humanized mouse models. Transfusion of human blood cells into cell-depleted NSG-MaFIA mice increased the persistence of the human cells in the circulation, indicating a role for monocytic cells in the removal of xenogeneic cells from immunodeficient mice.

## Introduction

Humanized mice are important for the study of human cells and preclinical research in general (1–3). The development of immunodeficient mouse strains that allow for the long-term engraftment of human cells enables the study of human cells under conditions that are often more physiologically normal than can be modeled *in vitro*. Most immunodeficient mouse strains currently used for xenotransplantation have a set of mutations that prevent the development of lymphocytes, thereby eliminating adaptive immune responses as well as preventing the clearance of foreign cells mediated by natural killer (NK) cells. One widely used strain of mice is the NOD.Cg-*Prkdc^scid^ Il2rg^tm1Wjl^*/SzJ (NSG) strain, which has been used in investigations of human hematopoietic cells and other cell types (4). The *Prkdc^scid^* mutation results in defective DNA repair causing the death of T and B lymphocytes during T-cell receptor or immunoglobulin chain recombination (5). Additionally, mutation of the common gamma chain of the interleukin (IL)-2 receptor (CD132) prevents cytokine signaling through receptors that use this receptor component, many of which are critical for lymphocyte development and survival (6). NSG mice also have additional genetic alterations that provide a more permissive environment for foreign cells (7). These include a deficiency in hemolytic complement as well as a range of maturational/functional defects that affect monocytic cells.

Despite the profound immunological defects in NSG mice, the fate of xenogeneic cells transferred to these mice are variable with cells from some species being rapidly rejected (7). Clearance of foreign cells also appears to be affected by their location. In the case of human hematopoietic engraftment, substantial levels of hematopoietic chimerism can be achieved in the bone marrow, whereas other peripheral hematopoietic tissues show lower levels of human chimerism (8), with the peripheral blood having the lowest levels (9). Despite extensive hematopoietic engraftment in the bone marrow, very few human red blood cells (RBCs) are found in the circulation. The brief survival time of human leukocytes and erythrocytes transfused into NSG mice points to mechanisms by which these cells are rapidly cleared from the circulation (10). A role for mouse macrophages in the clearance of human RBCs and platelets has been established based on macrophage depletion studies using clodronate liposomes (11, 12). However, it should be noted that a recent study challenged the long-held belief that clodronate liposomes only target macrophages by demonstrating that neutrophil functions, including phagocytosis, are also diminished by clodronate liposomes treatment (13).

Despite the maturational defects observed in NSG monocytic cells, attributed to the non-obese diabetic (NOD) genetic background (7), we have hypothesized that development of specific methods of depleting monocytic cells in NSG mice might allow for improved methods for the construction of humanized mice. Burnett et al. reported the development of C57BL/6J mice containing a macrophage Fas-induced apoptosis (MaFIA) transgene that enables drug-induced specific ablation of monocytic cells (14). The MaFIA gene construct contains the cytoplasmic domain of CD95/Fas linked to two copies of the FK506-binding protein and truncated human low-affinity nerve growth factor receptor (ΔLNGFR, ΔCD271), which anchors the gene construct to the cell membrane. Administration of the homodimerization agent, AP20187, results in dimerization of the FK506-binding proteins and activation of the CD95 domains inducing apoptosis. In addition to cell-surface expression of human ΔCD271, which can be used as a marker of MaFIA transgene expression, the MaFIA construct is bicistronic and contains the gene for enhanced green fluorescent protein (EGFP). Expression of the MaFIA gene construct is primarily targeted to monocytic cells using the *c-fms* promoter.

We report on the introgression of the MaFIA gene construct into the NSG mouse strain through 10 generations of breeding to NSG mice with offspring selected based on EGFP expression. A strain of NSG-MaFIA mice was developed with a functional MaFIA gene construct and the immunodeficiency of NSG mice. The survival of transfused human blood cells was tested in NSG-MaFIA mice after drug-induced cell depletion.

## Materials and methods

### Mice

Animal research was performed with approval and oversight of the Institutional Animal Care and Use Committee at Labcorp Early Development Laboratories Inc. (San Carlos, CA) under Animal Welfare Assurance A3367-01. Animal husbandry was carried out according to the recommendations in the Guide for the Care and Use of Laboratory Animals of the National Institutes of Health. All mice were housed in the same room under the same conditions. Mice were maintained in Good Laboratory Practices-certified sterile, disposable microisolator cages containing ALPHA-Dri bedding (Innovive Inc; San Diego, CA, USA) and fed a sterile, irradiated diet of Teklad Global 19% protein diet (Envigo) with free access to sterile-filtered, acidified water (Innovive Inc.). Mice were housed in groups of two to five mice to maintain normal social behavior unless behavioral problems or experimental necessity required individual housing. Environmental enrichment was provided including autoclaved cotton Nestlets (Ancare Corp.) and GLP-certified Bio-Huts (Bio-Serv) added to cages during fortnightly cage changes. Mice were exposed to an even 12-h light/dark cycle. Biannual nucleic acid testing was used to ensure that the vivarium remained free from >40 murine pathogens (Mouse Surveillance Plus PRIA; Charles River).

C57BL/6J (RRID: IMSR_JAX:000664), NSG (RRID: IMSR_JAX:005557), and C57BL/6-Tg(Csf1r-EGFP-NGFR/FKBP1A/TNFRSF6)2Bck/J (MaFIA; RRID: IMSR_JAX:005070) mice were obtained from the Jackson Laboratory, and colonies were bred and maintained at our institute. NSG-MaFIA mice were generated by crossing the NSG and MaFIA strains to generate F1 hybrids and backcrossing offspring with NSG mice for an additional nine generations (**Table 1**). Hybrid offspring used for breeding of each new generation were selected based on EGFP expression on peripheral blood cells as measured by flow cytometry, as described below. Hemizygous EGFP^+^ mice were selected from the N10 generation to form three breeder pairs. The F1 offspring from these breeder pairs were screened for EGFP expression levels to determine the genotype of the offspring with respect to the MaFIA transgene.

**Table 1:** Overview of breeding scheme used to generate NSG-MaFIA mice.

Generation	Male strain	Female strain	Offspring selection for next generation
P	MaFIA	NSG	Selected male mouse for breeding. No phenotyping performed.
N1	Hybrid N1	NSG	Selected female EGFP^+^ mouse for breeding: X-chromosomes with *Il2rg^tm1Wjl^* mutation of NSG origin.
N2	NSG	Hybrid N2	Selected male EGFP^+^ for breeding.
N3	Hybrid N3	NSG	Select male EGFP^+^ for breeding.
N4	Hybrid N4	NSG	Select male EGFP^+^ for breeding to refresh the NSG X-chromosome.
N5	Hybrid N5	NSG	Select female EGFP^+^ for breeding.
N6	NSG	Hybrid N6	Select male EGFP^+^ for breeding to refresh the NSG Y-chromosome.
N7	Hybrid N7	NSG	Select male EGFP^+^ for breeding.
N8	Hybrid N8	NSG	Select male EGFP^+^ for breeding.
N9	Hybrid N9	NSG	Select EGFP^+^ for brother X sister mating.
N10	Hybrid N10	Hybrid N10	Select EGFP^++^ offspring creating two potential founder pairs: A and B.
F1	F1	F1	Selected F1 Pair NSG-MaFIA-A as founders to establish NSG-MaFIA line based on analysis of F2 offspring.

Two F1 breeder pairs, NSG-MaFIA-A and NSG-MaFIA-B, that shared the N9 parentage were generated that were homozygous for the MaFIA transgene based on EGFP expression. Moreover, mice of the N10 generation that lacked EGFP expression or were hemizygous were used as controls in some experiments. Ultimately, the breeding pair NSG-MaFIA-A was selected to establish the NSG-MaFIA mouse colony based on confirmatory analysis of their offspring (F2). As the phenotype of the F2 mice generated from breeding pair NSG-MaFIA-B was very similar to those from pair A, as well as the shared 10-generational ancestry of the A and B lines, a limited number of NSG-MaFIA-B mice were used in experiments as indicated.

### Flow cytometry

EGFP expression and the expression of cell surface markers were analyzed by flow cytometry using either a BD LSR II (BD Biosciences, San Jose, CA, USA) or Cytek Aurora (Cytek Biosciences, Fremont, CA, USA) flow cytometers. Cell staining was performed as previously described (15). Propidium iodide (BioLegend, San Diego, CA, USA) or Live/Dead Blue (Life Technologies, South San Francisco, CA, USA) was used to stain dead cells. All phycoerythrin (PE), allophycocyanin (APC), PE-cyanine 7 (PE-Cy7), APC-cyanine 7 (APC-Cy7), PE-Fire 700, APC-Fire 750, Pacific Blue (PB), Alexa Fluor 594 (AF594), Alexa Fluor 647 (AF647), Alexa Fluor 700 (AF700), Brilliant Violet 421 (BV421), and Brilliant Violet 750 (BV750) labeled antibodies were purchased from BioLegend or an otherwise stated vendor. The following monoclonal antibodies were used to stain mouse cells: CD4 AF594 (clone GK1.5), CD8a BV750 (clone 53-6.7; BD Biosciences), CD19 AF700 (clone 1D3/CD19), CD45 PB (clone 30-F11), CD45 APC-Fire 750 (clone I3/2.3), CD45R/B220 APC-Cy7 (clone RA3-6B2), CD335 PE (clone 29A1.4), and T-cell receptor (TCR) β APC (clone H57-597). The following monoclonal antibodies were used to stain human cells: CD3 AF647 (clone OKT3), CD19 PE-Fire 700 (clone HIB19), CD14 PE (clone M5E2), CD45 PE-Cy7 (clone HI30), CD56 BV421 (clone 5.1H11), and CD235a PE (clone HI264).

Flow cytometry data were analyzed using FlowJo software 10 (FlowJo, Inc.; Ashland, OR, USA). Standard gating procedures included selection of live, single cells based on the lack of nucleic acid/cytoplasmic staining and light-scatter properties before additional gating was performed as described in the text. The percentages of CD45^+^ monocytes (low side-light scatter) and CD45^+^ granulocytes (high side-light scatter) in peripheral blood 1-day post drug treatment were determined by comparing with an untreated mouse of the same strain. Frequencies of live, single, CD45^+^, and EGFP^high^ peritoneal, bone marrow, and spleen cells were calculated in a similar fashion. Mean fluorescence intensities (MFIs) were compared using GraphPad Prism 10.4.1 software (GraphPad Software; San Diego, CA, USA). Data were charted showing individual values with a bar indicating the mean ± standard error of the mean. Ordinary one-way ANOVA with the Tukey’s multiple comparison test was used to evaluate the significance of differences among homozygous hybrids, hemizygous hybrids, and MaFIA mice for each tissue type. All strains of mice were n=3 with the exception of NSG-MaFIA-A, which were n=4. P< 0.05 was considered significant throughout this study. P-values are indicated in charts using the following categories: P<0.0001 (∗ ∗ ∗ ∗), P = 0.0001-0.001 (∗ ∗ ∗), P = 0.001-0.01 (∗ ∗), P = 0.01-0.05 (∗), and not significant (ns).

### Macrophage depletion in MaFIA mice

Mouse strains containing the MaFIA transgene were depleted of macrophages and dendritic cells (DCs) through the induction of apoptosis by daily intraperitoneal administration of 10 mg/kg AP20187 homodimerizer (Takara Bio USA, Inc) for 5 consecutive days per manufacturer’s instructions and as previously described (16). Briefly, lyophilized AP20187 was dissolved in 100% ethanol at a concentration of 62.5 mg/mL stock solution and stored at −20°C. For *in vivo* use, peritoneal injections were prepared from stock solution by dilution in polyethylene glycol 400 and 2% Tween-20 in deionized water. All injections were administered within 30 min of dilution.

Mouse weight data were normalized to 100%, defined by the first measurement made immediately before the first injection of homodimerizer. Ordinary one-way ANOVA with Tukey’s multiple comparison test was used to evaluate significant differences. All error bars in charts represent the standard error of the mean.

### Complete blood counts

Complete blood counts (CBCs) were determined using a Heska Element HT5 (Loveland, CO, USA) hematology analyzer. A 20-μL sample of whole blood was collected from the tail tip of mice into ethylenediaminetetraacetic acid Minivettes from Sarstedt (Nümbrecht, Germany).

### Cell isolation and processing

Blood was collected for CBCs and flow cytometry as previously stated (10). Mice were euthanized by carbon dioxide asphyxiation followed by cervical dislocation. Peritoneal cells were flushed from the peritoneal cavity with 10 mL of ice-cold phosphate-buffered saline (PBS) using a syringe with a 16-gauge needle (Becton Dickinson; NJ, USA). The bloated cavity was gently massaged and as much PBS as possible was drawn back into the syringe before piercing through the opposite side of the peritoneal wall to allow for more of the remaining fluid to drain into a 15-mL conical tube (17). Central bone marrow was collected from femurs by flushing with ~1.5 mL of PBS using a 27-gauge needle (Becton Dickinson; NJ, USA) (18). Whole spleens and liver were isolated and kept in PBS until cell isolation. All tissues were held on ice after harvest.

Leukocytes were enriched from peripheral blood by the addition of ACK (ammonium chloride potassium) lysing buffer (Gibco; NY, USA) followed by a 5-min incubation at room temperature in the dark. Cells were centrifuged for 5 min at 300 × g at 4°C and washed with the same settings. Peritoneal and bone marrow cells were filtered through a 100-μm strainer (Greiner Bio-One; NC, USA) and washed by centrifugation for 5 min at 300 × g at 4°C. Cell suspensions were prepared from spleens and livers by passage through a 100-μm strainer using a 5-mL syringe plunger (Becker Dickinson; NJ, USA). Cells were washed by centrifugation. All cells were suspended in blocking buffer for staining with fluorochrome conjugated mAbs (15). Twice-washed cells were analyzed by flow cytometry the following day or fixed so that analysis could be performed in subsequent days.

### Measurement of radiation sensitivity

Mice were fed a sterile, irradiated diet of Teklad 18% protein extruded diet (Inotiv) exchanged for a Teklad 4,100 ppm Uniprim diet (Inotiv) 1 week prior to radiation. X-ray irradiation was delivered using an RS 2000 irradiator (RadSource; Buford, GA, USA). Mice were irradiated with either 175 or 700 cGy. Survival was analyzed using Kaplan–Meier analysis (GraphPad Prism 10.4.1). Data from multiple groups of mice are presented as the percentage of individuals that survive in each group over time. The Mantel–Cox test was used to compare multiple survival curves using Bonferroni’s method to correct for multiple comparisons. A Bonferroni-corrected α-value of 0.0167 was calculated with the family-wise alpha level value of 0.05 and a K-value for number of comparisons as 3. P-values of <0.0167 were considered significant for survival statistics.

### Enzyme-linked immunosorbent assay

Serum was collected from mice as previously reported (19) and was frozen at −80°C until needed for analysis. Murine hemolytic complement factor 5 (C5) in the serum was measured using an enzyme-linked immunosorbent assay (ELISA) kit purchased from Biomatik (Delaware, USA). The assay was conducted according to the manufacturer’s protocol, but a 1:100 instead of the recommended 1:200 serum dilution was used. Absorbance readings were made at 450 and 570 nm using a CLARIOstar Plus (BMG Labtech; Ortenberg, Germany). Subtraction of 570-nm readings from 450-nm readings were made for correction of optical imperfections in the plate.

### Sanger sequencing

Total genomic DNA was prepared from 1-2-mm slices of tails using KAPA Mouse Genotyping Kit (Roche, USA). A 147-bp product containing a portion of the C5 gene (*Hc*) was amplified using the following primer set: forward: TCCACAGGTATGGTGTTTGG and reverse: CCAAAGGTACTGCAAAATCC. Polymerase chain reaction (PCR) products were sent to GENEWIZ (Azenta Life Sciences, USA) for sequencing. Benchling (USA) was used to map DNA sequences to “Mouse complement component C5D (pro-C5D) mRNA, complete cds” (GenBank Accession M35526.1 (20)) as a reference. Sanger sequencing results were analyzed using Benchling’s (USA). The multi-sequence MAFFT alignment program was set to “auto”. GenBank Accession M35526.1 was used as the reference sequence for alignment.

### Transfusion of human RBCs

Human blood cells were obtained from volunteer blood-donors (Vitalant). These samples did not meet the definition of human subjects research as the blood was not collected specifically for experimental purposes and the donors were anonymous. Leukoreduced blood was prepared from peripheral blood using Acrodisc PSF 25mm white blood cell filters (AP-4952; Pall Corporation; NY, USA), which was used as a source of RBCs. These RBCs were stored overnight in PBS at 4°C, and 100 μL was transfused into NSG, MaFIA, and NSG-MaFIA mice the next day. Blood was collected via the tail into PBS + 14% citrate phosphate dextrose adenine solution and analyzed by flow cytometry to measure human CD235a (hCD235a) expression (10). A second experiment compared RBC transfusion in NSG and macrophage-depleted NSG-MaFIA mice.

Frequency data for hCD235a^+^ events in peripheral blood at various times after transfusion were normalized based on the first measured time point, at 2 min, which was defined as 100%. Data normalization was used to reduce variability associated with differences in size of the mice and number of transfused RBCs (10). The two-way ANOVA method with Sidak’s multiple comparison test was used for analysis of engraftment. Area under the curve analysis was also employed to compare relative amounts of engraftment between strains.

### Human peripheral blood cell engraftment

Male mice were transplanted with human leukocytes similar as previously described (21, 22). Human leukocytes were prepared from peripheral blood by filtration using Acrodisc PSF 25-mm white blood cell filters followed by back-flushing the filters to recover the leukocytes (23). CBCs were performed to obtain cell counts. NSG-MaFIA were pretreated with a 5-day course of homodimerizer agent prior to human cells transfusion. Untreated NSG mice were transfused with human cells as controls.

In the first experiment, mice were transplanted with 1.3 × 10^7^ leukocytes, and in the second experiment, this number was reduced to 1 × 10^6^ leukocytes. Leukocytes were held overnight in human serum, from AB-blood antigen^+^ donors (Valley Biomedical; VA, USA) at 4 °C, and transfused the next day. Survival analyses are shown using Kaplan–Meier analysis; animals euthanized for engraftment analysis were censored. Mice were analyzed for human cell chimerism after 2 and 3 weeks. Terminal blood collections were used to analyze the final blood sample as described (19). Central femoral bone marrow, spleen, and liver were also collected for analyses. Light-density cell separation was used to enrich human mononuclear cells from cell and tissue specimens. Total cell counts were determined using a Cellometer K2 (Revvity; MA, USA). The total number of light-density bone marrow cells per animal was calculated by estimating that two femora contain 13% of all bone marrow cells (18). The percentages or total number of hCD45^+^ events among live, single-cell, total blood leukocytes at 7, 14, or 20 days after transfusion were compared using a two-tailed unpaired t-test.

## Results

### Introduction of the MaFIA transgene into NSG mice

**Figure 1:**
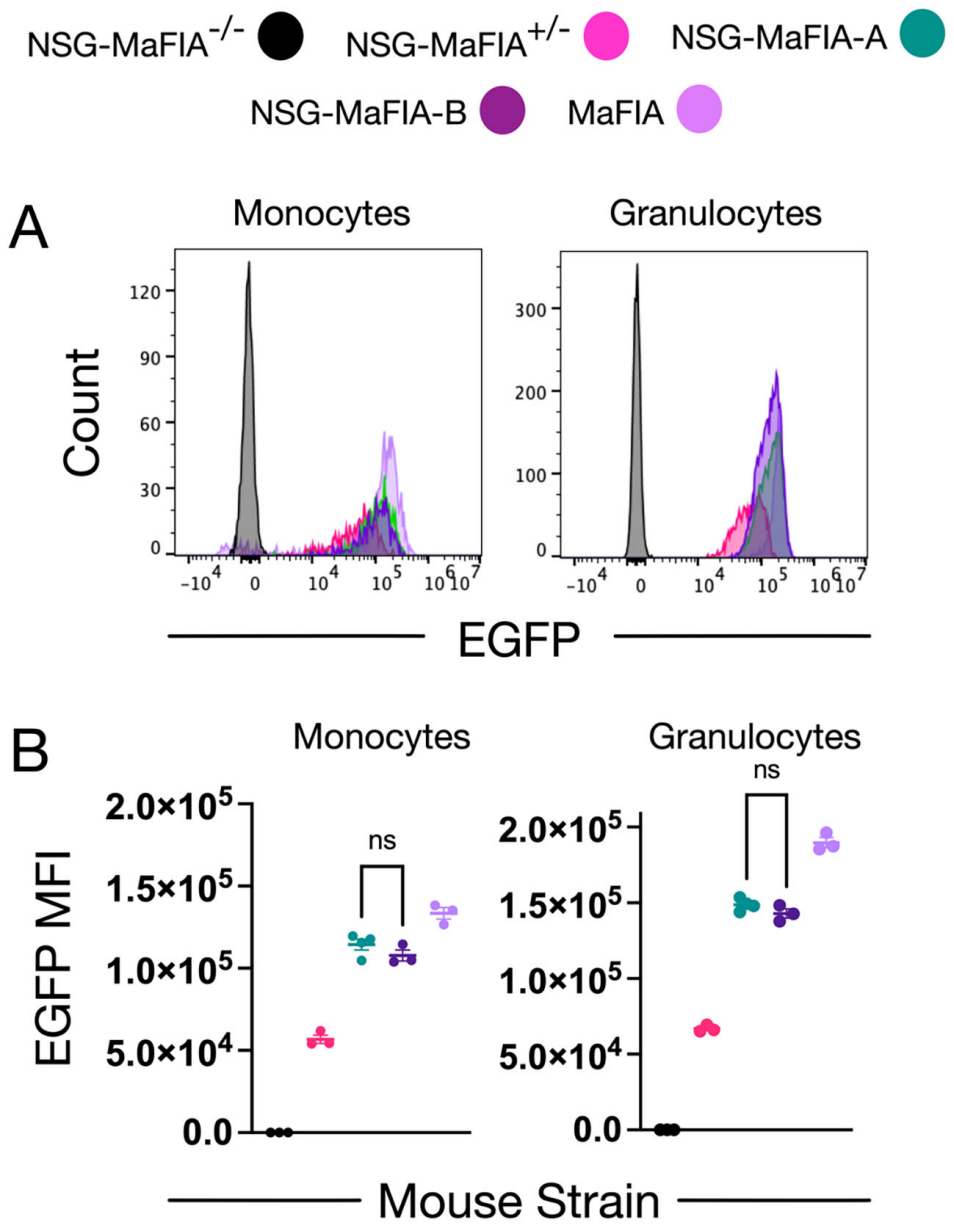
Selection of NSG-MaFIA mice by EGFP expression analysis. **(A)** Comparison of EGFP fluorescence from peripheral-blood monocytes and granulocytes from NSG mice homozygous for the MaFIA transgene (NSG-MaFIA-A and NSG-MaFIA-B), hemizygous for the transgene (NSG-MaFIA^+/−^), and hybrid mice lacking the transgene (NSG-MaFIA^−/−^). EGFP fluorescence from cells obtained from a MaFIA mouse is shown for comparison. Histograms represent live-single CD45^+^ cells gated for cells expressing low (monocytes) and high (granulocytes) levels of side-light scatter. **(B)** EGFP MFI for MaFIA and NSG-MaFIA hybrid mice. Each symbol represents data from a single mouse. For both cell types, all groups differ significantly except for the one comparison shown labeled not significant (ns).

Introduction of the MaFIA transgene construct (14) was accomplished by 10 generations of crosses to NSG mice (**Table 1**) with offspring selection based on detection of EGFP fluorescence on peripheral blood monocytes and granulocytes (**Figure 1A**). The design of the breeding scheme insured homozygosity of the *Il2rg* mutation, found on the X-chromosome, in the N2 generation. Two breeder pairs of congenic founders were created, NSG-MaFIA-A and NSG-MaFIA-B. Comparisons of EGFP MFI demonstrated the same levels of expression from the NSG-MaFIA-A and NSG-MaFIA-B lines (**Figure 1B**). However, these expression levels were 1.2- to 1.3-fold higher in the parental MaFIA mouse strain (P<0.0001). EGFP fluorescence was affected by gene dosage with the hemizygous mice having mean MFI values that were 45.0% to 52.7% of the values from the homozygous mice (P<0.0001).

### Confirmation of a functional MaFIA transgene in NSG-MaFIA mice

**Figure 2:**
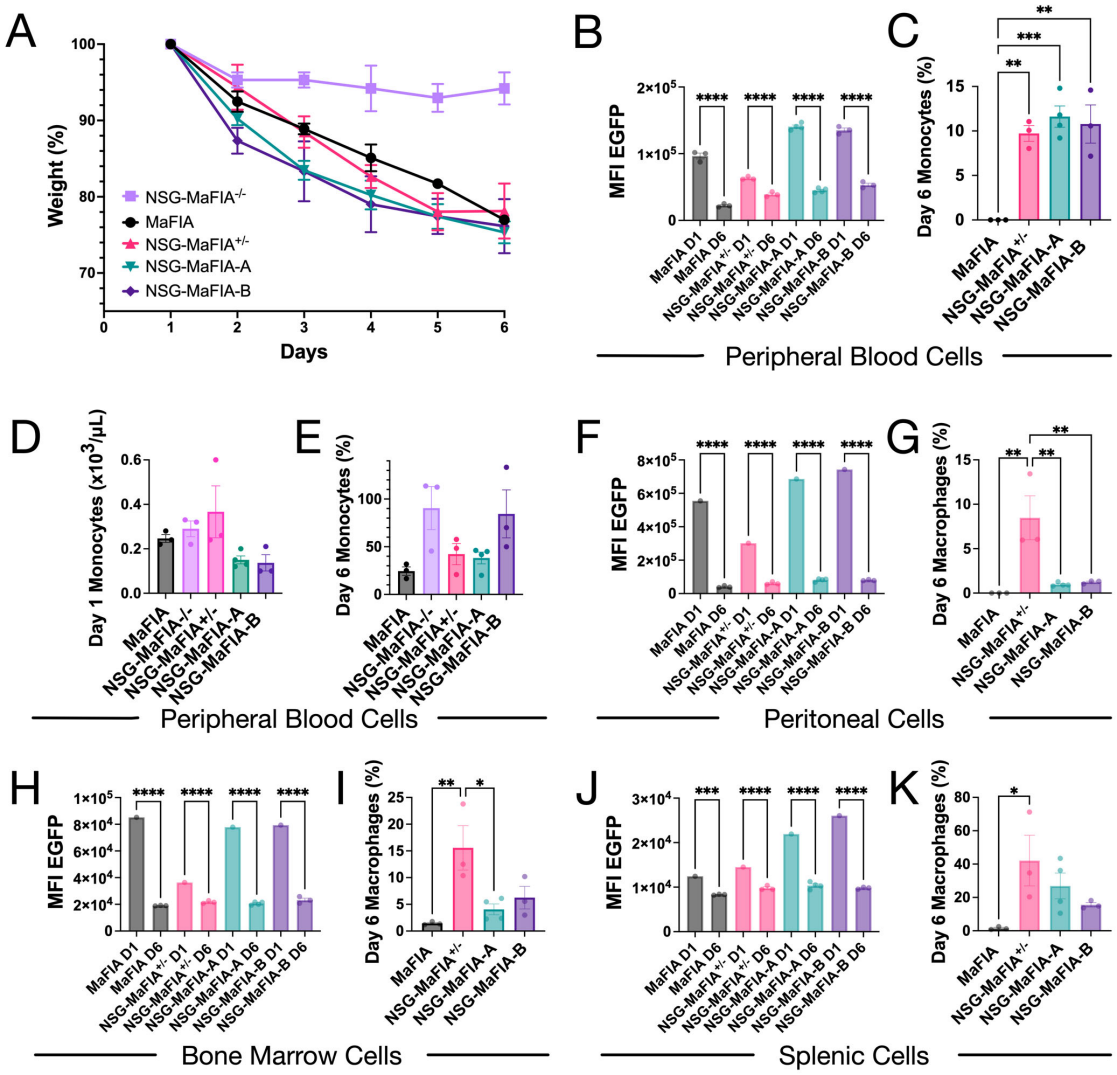
The MaFIA transgene is functional in NSG-MaFIA mice. **(A)** Normalized mouse weights immediately before (day 1, D1), during, and 1 day after (day 6, D6) AP20187 homodimerizer treatment. EGFP MFI for CD45^+^EGFP^+^ peripheral blood **(B)**, peritoneal **(F)**, bone marrow **(H)**, and splenic **(J)** cells for untreated (D1) and treated (D6) mice. Flow cytometric analysis of CD45^+^EGFP^+^ monocytes in peripheral blood **(C)** and macrophages in the peritoneum **(G)**, bone marrow **(I)**, and spleen **(K)** on day 6 as a percentage of day 1 or untreated measurements. Gating used to define monocytes and macrophages are shown in **Figures 3B–D**. Monocyte counts from CBCs on day 1 **(D)** and day 6 counts as a percentage of day 1 counts **(E)**.

To determine if a functional MaFIA transgene was successfully incorporated into the new mouse lines, F2 mice were treated for 5 days with an AP20187 homodimerizer (14). Both lines of NSG-MaFIA mice and MaFIA mouse controls lost on average 23.85 ± 1.88% of their body weight after the 5-day treatment (**Figure 2A**). Interestingly, hemizygous NSG-MaFIA^+/−^ mice lost a comparable amount of weight to homozygous mice. There were no significant differences, at any time points, among the MaFIA homozygous or hemizygous lines. It should be noted that homodimerizer-treated NSG mice lost 5.8 ± 2.1% of their body weight, with most of the weight loss happening after the first day of treatment.

**Figure 3:**
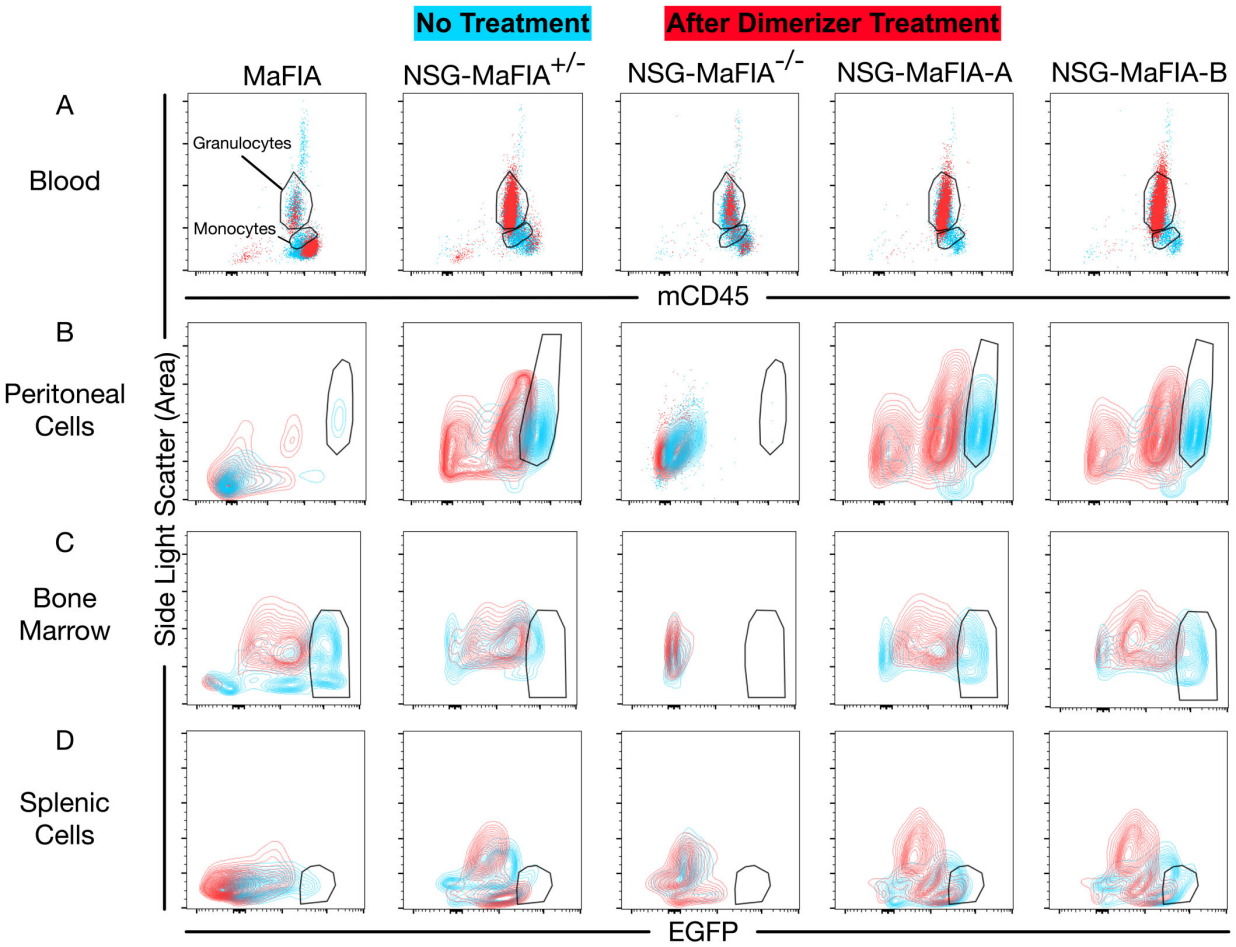
Flow cytometric analysis of homodimerizer-induced cell depletion. Representative flow cytometry plots are overlayed from untreated mice (blue) and mice evaluated on day 6, one day after final homodimerizer treatment (red). All data show side-light scatter, on a linear scale, to differentiate granulocyte and monocytic cell populations from other blood cell types. **(A)** Whole-blood cells were gated to show mouse CD45^+^ (mCD45) leukocytes, and monocyte and granulocyte gates were drawn that capture most EGFP expression, as per **Figure 1**. EGFP expression is shown for CD45^+^ peritoneal cells **(B)**, bone marrow cells **(C)**, and splenocytes **(D)**. CD45 and EGFP fluorescence data are biexponentially transformed for display. Gates on the monocytic cell populations **(B-D)** are shown as a visual aid to highlight the loss of cells expressing high levels of EGFP after homodimerizer treatment.

One day after the final homodimerizer treatment, the MFI of EGFP expression and the frequencies of monocytes in blood were analyzed by flow cytometry (**Figure 3A**). The levels of EGFP expression were measured on total EGFP^+^ cells, thereby minimizing the influence of EGFP^−^ lymphocytes found only in MaFIA mice. Homodimerizer treatment decreased EGFP expression in all mice (**Figure 2B**). The frequencies of CD45^+^EGFP^+^ monocytes decreased after the drug treatment by about 90% in the peripheral blood in mice with an NSG genetic background, but monocyte levels were still significantly greater relative to the near-complete reduction seen in MaFIA mice (**Figure 2C**). Based on CBC analysis, the number of monocytes in the blood before (**Figure 2D**) and after (**Figure 2E**) dimerizer treatment did not differ significantly among the mouse strains. CBC results presented greater variability compared with flow cytometric analyses, which uses more than just light-scatter parameters to define monocytes.

The loss of monocytic cells in different tissues was similarly examined after homodimerizer treatment. In the peritoneal cavity, which normally has a population of resident macrophages (24), the MFI of EGFP expression on CD45^+^ cells significantly decreased after homodimerizer treatment (**Figure 2F**). The EGFP MFI decrease was readily observed as a loss of cells expressing the highest levels of EGFP and an increase in the number of cells expressing low levels of EGFP or lacking EGFP expression (**Figure 3B**). Unlike the pattern observed with peripheral blood monocytes, the depletion of peritoneal macrophages, defined by the gating shown in **Figure 3B**, was greater in the homozygous NSG-MaFIA-A and NSG-MaFIA-B mice than in the hemizygous mice (**Figure 2G**).

It is possible that the depletion of macrophages in the peritoneum was influenced by exposure to a high concentration of homodimerizer, as this drug was delivered by intraperitoneal injection. Indeed, monocytic depletion in peritoneal space was greater in magnitude than observed in spleen or bone marrow (**Figure 2**). Monocytic cells in the bone marrow (**Figure 2I**) and spleen (**Figure 2K**) were generally more effectively depleted in homozygous mice than in the hemizygous mice in contrast to what was observed in the blood. Significant reductions in EGFP MFI in the bone marrow (**Figures 2H**, **3C**) and spleen (**Figures 2J**, **3D**) also followed the same pattern as observed in the peritoneum. Overall, monocytic cells in the hemizygous mice were depleted by ≥90% in the peripheral blood (**Figure 2C**) and peritoneal cavity (**Figure 2G**), but only about 85% in bone marrow (**Figure 2I**), and 60% in the spleen (**Figure 2K**). Despite comparable weight loss exhibited by hemizygous NSG-MaFIA^+/−^ mice and homozygous NSG-MaFIA^+/+^ mice, the depletion of macrophages in tissues was greater in the homozygous mice. Thus, both the NSG-MaFIA-A and NSG-MaFIA-B lines have a functional MaFIA gene construct enabling the depletion of monocytic cells.

### NSG-MaFIA mice are lymphopenic

Flow cytometric comparison of blood cell populations before and after homodimerizer treatment shows a relative enrichment of granulocytes after homodimerizer treatment in immunodeficient mice containing the MaFIA gene-construct compared with NSG mice lacking the construct (**Figure 3A**). In MaFIA mice, the depletion of monocytes led to a noticeable enrichment of low side-light scatter cells (lymphocytes), which was not observed in immunodeficient mice lacking lymphocytes.

**Figure 4:**
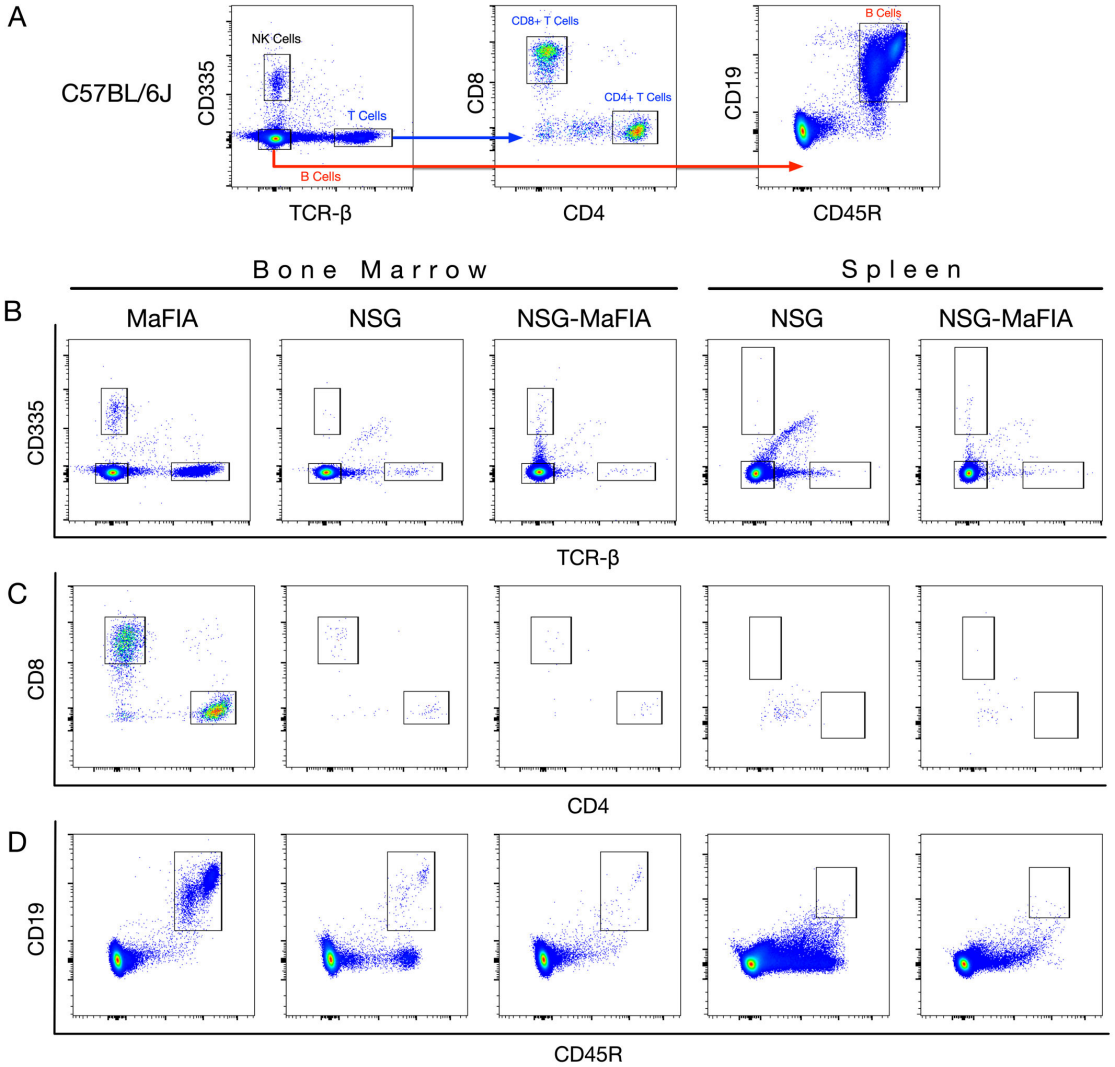
NSG-MaFIA mice are deficient of lymphocytes. **(A)** Flow cytometric analysis of NK, T, and B cells performed using immunocompetent C57BL/6J bone marrow. **(B)** Analysis of CD335^+^ NK cells and TCR-β^+^ T cells in the bone marrow and spleen of immunocompetent MaFIA, an immunodeficient NSG, and NSG-MaFIA-A mice. Analysis of the same tissues for the presence of CD4^+^ and CD8^+^ single-positive T cells out of all TCR-β^+^ T cells **(C)** and CD19^+^CD45R^+^ B-cells out of all CD335^-^TCR-β^−^ events, as shown in A **(D)**.

Immunodeficiency of the NSG-MaFIA mice was also examined by CBC measurements and flow cytometry (25). CBC measurements from various strains of mice showed that MaFIA mice have 9.78 × 10^3^ lymphocytes/μL whereas NSG-MaFIA-A and NSG had over 120-fold fewer lymphocytes (P-values <0.0001; **Supplementary Table 1**). Flow cytometric analyses showed that NSG-MaFIA-A exhibited severe lymphopenia similar to NSG mice (**Figure 4**). The NSG-MaFIA mice had significantly lower frequencies of CD335^+^ NK-cells (**Figure 4B**), TCR-β^+^CD4^+^ T-cells (**Figure 4C**), TCR-β^+^CD8^+^ T-cells (**Figure 4C**), and CD19^+^CD45R^+^ B cells (**Figure 4D**) in the bone marrow and spleen compared with the immunocompetent strains.

### NSG-MaFIA mice are sensitive to X-ray irradiation

**Figure 5:**
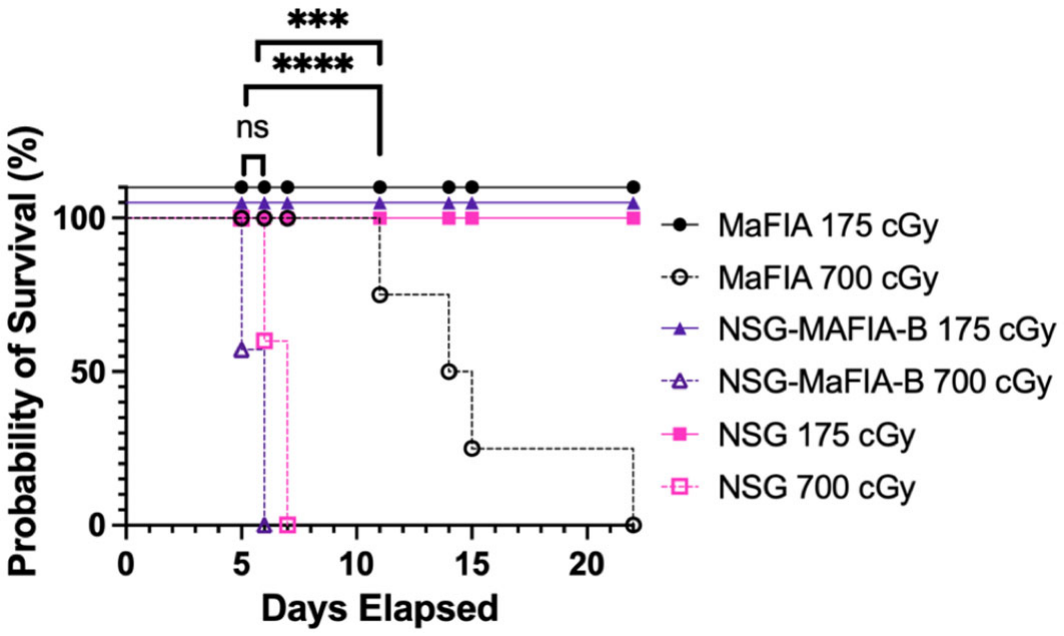
NSG-MaFIA mice are radiation sensitive. Kaplan–Meier survival curves comparing survival of three strains of mice at 175 and 700 cGy of X-ray radiation (n = 5 mice/group). Groups are staggered to visually separate overlapping data points. P<0.0001 (∗ ∗ ∗ ∗) and P = 0.0001-0.001 (∗ ∗ ∗).

Homozygous *Prkdc^scid^* mutation results in an increased sensitivity to ionizing radiation (5). The sensitivity of NSG-MaFIA-B mice to X-ray irradiation at 175 and 700 cGy was tested to determine if the *Prkdc^scid^* mutation found in NSG mice was present in the hybrid NSG-MaFIA strain. All mice that received the 175-cGy dose survived and did not exhibit any signs of abnormalities (**Figure 5**). However, all mice succumbed to 700-cGy radiation, with NSG-MaFIA-B mice having the same sensitivity to this dose as NSG mice, whereas MaFIA mice survived significantly longer.

### NSG-MaFIA mice have a mutated C5 gene

**Figure 6:**
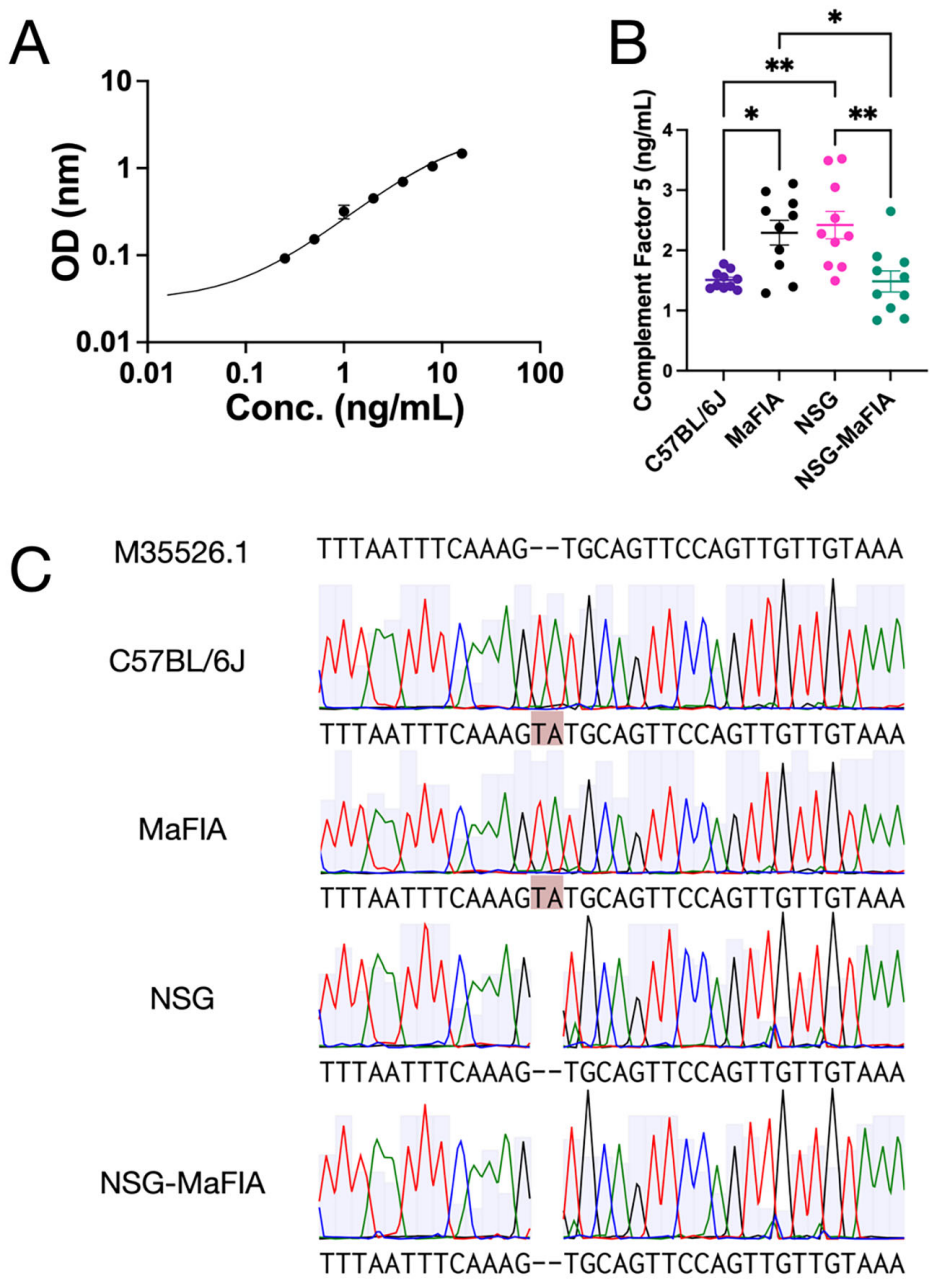
NSG-MaFIA mice have a mutated *Hc* gene. **(A)** C5 standard curve as determined by ELISA (n=2). **(B)** Serum concentrations of C5 from C57BL/6J (n=10), MaFIA (n=10), NSG (n=10), and NSG-MaFIA-A mice (n=10). **(C)** Representation of PCR and Sanger sequencing-based analysis of the *Hc* gene displaying a 2-bp deletion in C57BL/6J, MaFIA, NSG, and NSG-MaFIA-A mice. P = 0.001-0.01 (∗ ∗) and P = 0.01-0.05 (∗).

To determine if C5 functional deficiency was carried over into NSG-MaFIA mice, circulating levels of C5 were measured and DNA sequencing for the mutation was undertaken. The ELISA revealed that NSG-MaFIA-A mice contained significantly less C5 protein in comparison with NSG and MaFIA mice (**Figures 6A, B**), but the levels were comparable with those seen in C57BL/6J mice. Sequencing of genomic DNA further revealed a 2-base pair “TA” deletion in the *Hc* gene, which encodes C5, of NSG background mice (**Figure 6C**). This deletion introduces a termination codon 4-bp downstream, causing C5 functional deficiency (20). A similar 2-bp deletion was observed in NSG-MaFIA mice and other mouse strains with a 2-bp TA deletion in C5 deficient mouse (GenBank Accession M35526.1) but not in immunocompetent MaFIA and C57BL/6J mice (**Figure 6C**) (20).

### Prolonged circulation of human erythrocytes in macrophage-depleted NSG-MaFIA mice

**Figure 7:**
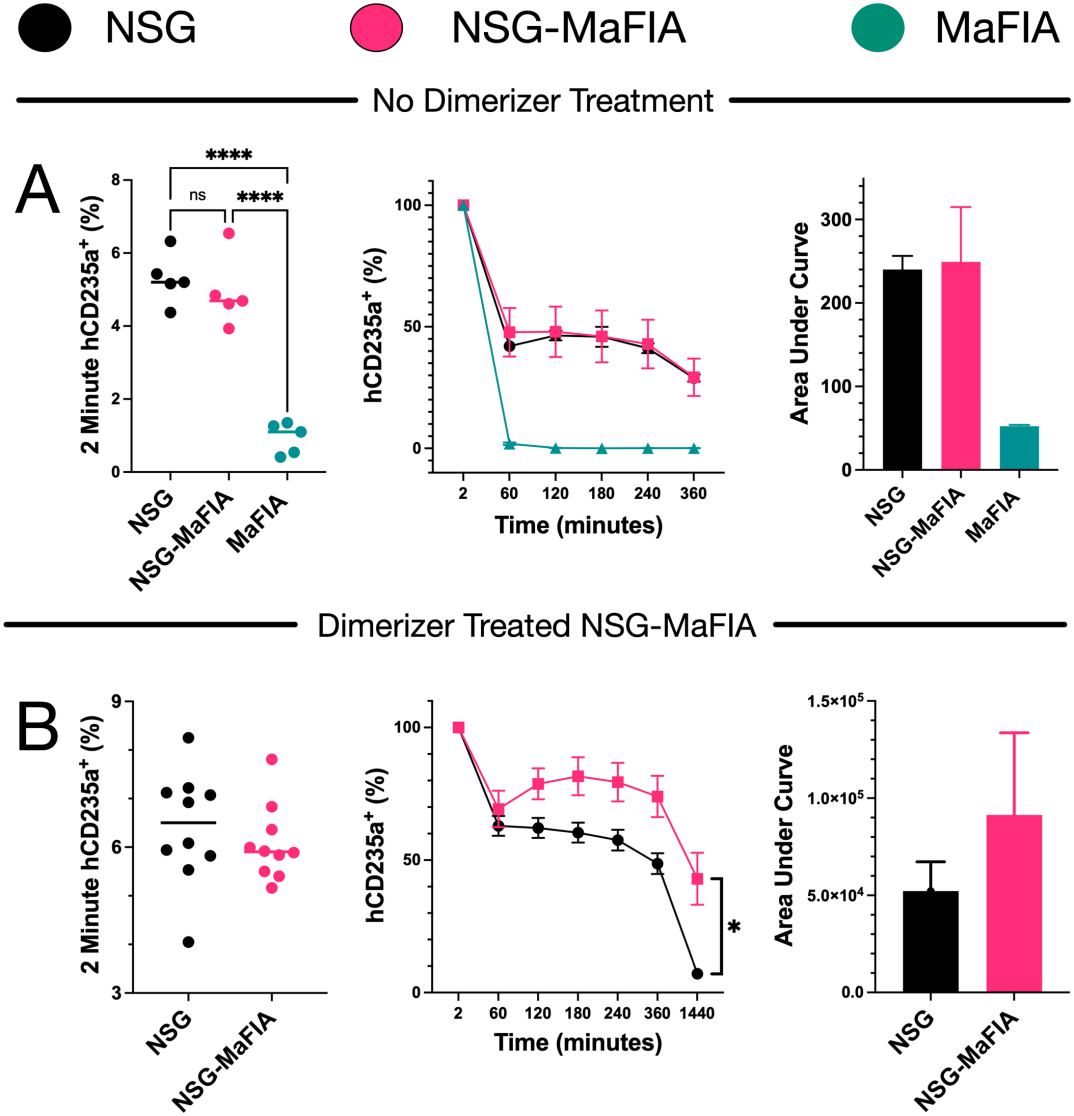
Clearance of transfused human erythrocytes in NSG-MaFIA mice. **(A)** Frequencies of hCD235a^+^ events, among all events, in the blood of MaFIA, NSG, and NSG-MaFIA-B mice after transfusion (n = 5 mice per group). Frequencies of hCD235a^+^ out of total hCD235a^+^ measured at 2 min after transfusion (middle). Area under curve analysis is shown alongside frequencies as a representation of total amount of engraftment over 6 h (right). **(B)** Human red blood cell frequencies and area under the curve analysis after human erythrocyte transfusion into NSG mice and NSG-MaFIA-A mice one day after final homodimerizer treatment (n = 10 mice per group). P<0.0001 (∗ ∗ ∗ ∗) and not significant (ns).

As depletion of macrophages using clodronate liposomes is known to increase the circulation time of human RBCs in NSG mice (11), human RBC transfusion was used to evaluate the NSG-MaFIA mice. Even without macrophage depletion, the deficiencies of NSG mice allow for longer circulation of human RBCs than observed in immunocompetent mouse strains (10). Indeed, a rapid loss of circulating human RBCs was observed in MaFIA mice, even after only 2 min, compared with NSG and NSG-MaFIA-B mice (**Figure 7A**; strain effect P = 0.0002). Comparisons of NSG and untreated NSG-MaFIA-B mice at different time points observed no significant differences between these two strains.

Transfusion of human RBCs into homodimerizer-treated NSG-MaFIA-A mice resulted in prolonged circulation of the human cells (**Figure 7B**; strain effect P = 0.0174). Prior to homodimerizer treatment, both groups of mice had similar mean weights: 27.6 ± 0.4 g for NSG-MaFIA and 27.5 ± 1.2 g for NSG mice (n = 10 each). On the day of transfusion, NSG-MaFIA mice weighed 20.8 ± 0.3 g, a 24.5% decline. Nonetheless, the frequencies of RBCs at the initial measurement were not significantly different between the two mouse strains (**Figure 7B**). Area under the curve analysis over 24 h indicated a 1.75-fold increase in circulating human RBCs in the NSG-MaFIA mice.

### Enhanced engraftment of human leukocytes in macrophage-depleted NSG-MaFIA mice

**Figure 8:**
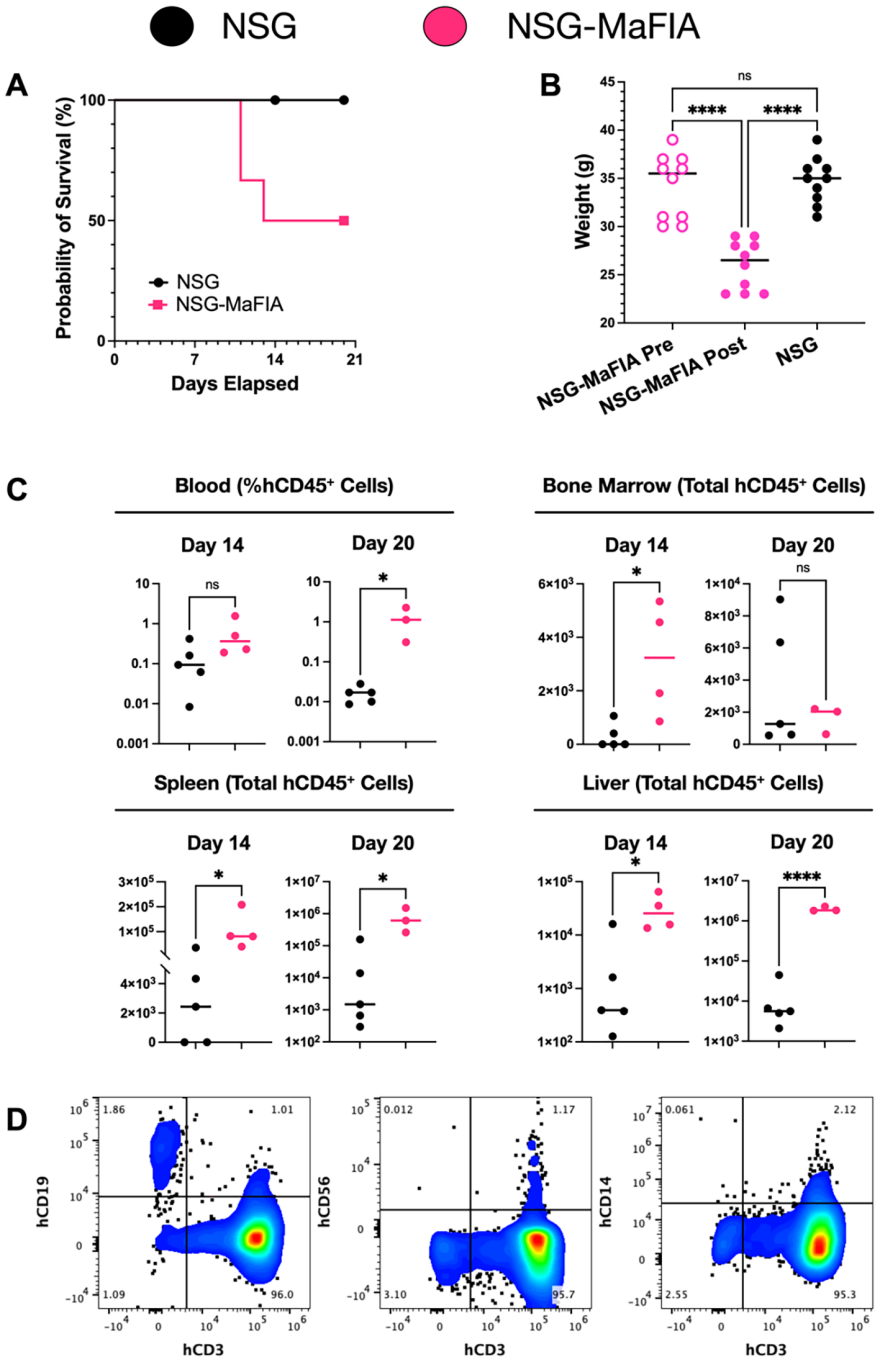
Human leukocyte engraftment in NSG-MaFIA mice. **(A)** Kaplan–Meier plot showing survival after transfusion with 1.3 × 10^7^ human leukocytes. **(B)** Mouse weights prior to homodimerizer treatment of NSG-MaFIA mice and at the time of leukocyte transfusion, one day after final homodimerizer treatment. **(C)** Frequencies of human blood leukocytes and the total numbers of human CD45^+^ cells in the indicated tissues at 14 and 20 days after transfusion. **(D)** Flow cytometric analysis of light-density spleen cells of an NSG-MaFIA mouse 20 days after transfusion of human cells. Note the scales on the y-axes are not consistent between graphs and alter between linear and logarithmic scales to best display the data. P<0.0001 (∗ ∗ ∗ ∗), P = 0.01-0.05 (∗), and not significant (ns).

As comparison of the NSG-MaFIA-A and NSG-MaFIA-B mice did not reveal any notable or consistent differences between these two lines, the NSG-MaFIA-A line was selected and is, hereafter, referred to simply as NSG-MaFIA. To follow-up on the observation that human RBCs have a prolonged circulation time in homodimerizer-treated NSG-MaFIA mice, these mice were further tested for susceptibility to engraftment by human leukocytes. NSG and homodimerizer-treated NSG-MaFIA mice (n = 10 each group) were transfused with 1.3 × 10^7^ human leukocytes. No NSG mice died during the 20-day observation period, but three NSG-MaFIA mice died between days 11 and 13 after transfusion (**Figure 8A**). At the end of the homodimerizer treatment, the NSG-MaFIA mice had lost weight and were lighter, as a group, than the control NSG mice (**Figure 8B**).

**Figure 9:**
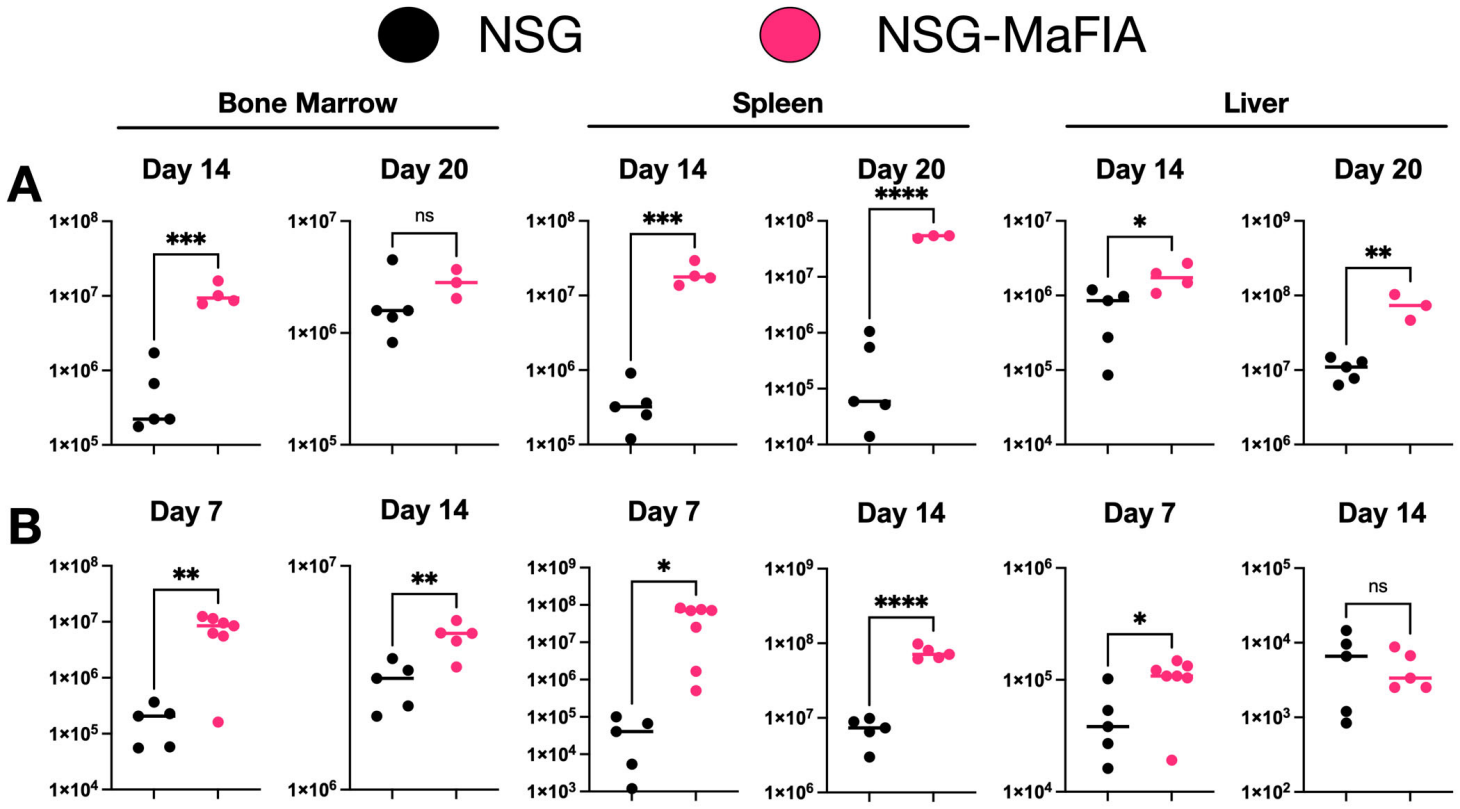
Tissue cellularity after human leukocyte engraftment. **(A)** Total tissue cell numbers at 14 and 20 days after transfusion into NSG and NSG-MaFIA mice with 1.3 × 10^7^ human leukocytes. **(B)** Cell numbers at 7 and 14 days after transfusion with 1.0 × 10^6^ human leukocytes. Note that cell numbers on the y-axis are shown on different logarithmic scales. P<0.0001 (∗ ∗ ∗ ∗), P = 0.0001-0.001 (∗ ∗ ∗), P = 0.001-0.01 (∗ ∗), P = 0.01-0.05 (∗), and not significant (ns).

Two weeks after transfusion, mice were analyzed for human engraftment. Although there was a 4.1-fold higher mean frequency of human leukocytes in the blood of NSG-MaFIA mice on day 14, the difference was not significant (**Figure 8C**). By 20 days after transfusion, the difference in blood leukocytes became 77-fold higher and was significant. Total cell numbers were, in nearly all cases, significantly elevated in the bone marrow, spleen, and liver in NSG-MaFIA mice on days 14 and 20 (**Figure 9A**). Likewise, the total number of CD45^+^ cells found in these tissues was significantly higher in NSG-MaFIA mice than in NSG mice with the exception of the bone marrow on day 20 (**Figure 8C**). Additional phenotyping of the engrafted human cells revealed that nearly all of these cells were CD3^+^ T-cells (**Figure 8D**). A small proportion of CD19^+^ B cells was observed in some tissues with higher levels of engraftment.

**Figure 10:**
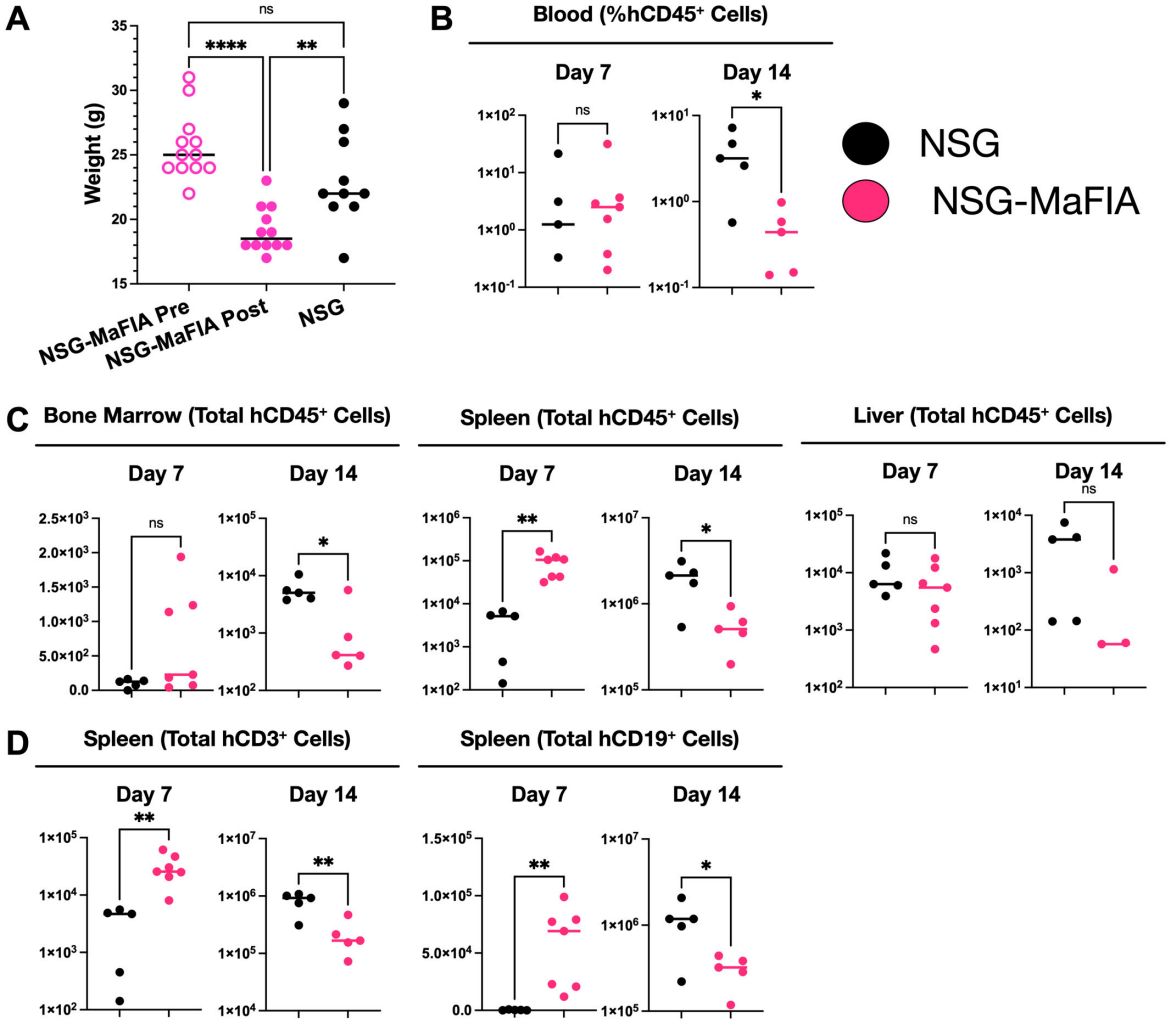
Human leukocyte engraftment in NSG-MaFIA mice after a low-dose transfusion. **(A)** Mouse weights prior to homodimerizer treatment of NSG-MaFIA mice and at the time of transfusion with 1.0 × 10^6^ human leukocytes. **(B)** Frequencies of human blood leukocytes at 7 and 14 days after transfusion. **(C)** Total numbers of human CD45^+^ cells in the indicated tissues at 7 and 14 days after transfusion. **(D)** Total numbers of human CD3^+^ T cells and CD19^+^ B cells in the spleen at 7 and 14 days after transfusion. Note the scales on the y-axes are not consistent between graphs and alter between linear and logarithmic scales to best display the data. P<0.0001 (∗ ∗ ∗ ∗), P = 0.001-0.01 (∗ ∗), P = 0.01-0.05 (∗), and not significant (ns).

A second experiment was performed with a reduced number of donor cells, 1.0 × 10^6^ human leukocytes, to delay engraftment and the onset of xenogeneic graft-versus-host disease which was suspected to contribute to the demise of some mice in the first experiment (22). Engraftment was analyzed earlier, at 7 and 14 days, and no animals died prior to analysis. Weight loss after homodimerizer treatment was observed as expected (**Figure 10A**). Both groups had a similar frequency of human leukocytes in the blood after 7 days, but at 14 days, the frequency of leukocytes decreased significantly in NSG-MaFIA mice (**Figure 10B**). The numbers of mouse and human cells recovered from NSG-MaFIA mice was significantly higher than from NSG mice with the exception of liver cells at 14 days (**Figure 9B**). The total number of human leukocytes was significantly higher in the spleen and was the site with the greatest number of engrafted human cells at 7 days in NSG-MaFIA mice (**Figure 10C**). Although the number of human leukocytes in the spleen increased further by day 14 in NSG-MaFIA mice, this increase was not as large as in NSG mice, and engrafted human cells were significantly lower in the spleen and bone marrow of NSG-MaFIA mice. As in the first experiment, the human leukocytes were composed dominantly of T and B cells, with higher proportions of B cells observed in the spleen of the second experiment (**Figure 10D**).

## Discussion

A line of NSG-MaFIA mice was generated through breeding that resulted in the introduction of the MaFIA transgene into the NSG genetic background. The resulting line of mice was selected based on EGFP expression by myeloid cells. Selective depletion of blood monocytes and cells expressing high levels of EGFP in hematopoietic tissues was demonstrated after AP20187 homodimerizer administration. We have also confirmed depletion of splenic CD11c^+^CD26^+^ DCs and CD11b^+^F4/80^+^ macrophages by homodimerizer treatment (JQ Tran, EJ Du, G Rivera-Torruco, AJ Hui, K Luong, S House, C Tse, A Tam, RP Jackman, MO Muench, manuscript submitted). Key phenotypic properties of the NSG strain of mice were maintained in NSG-MaFIA mice: a lack of lymphocytes, radiation sensitivity, and C5 functional deficiency.

The NOD genetic background introduced an altered monocytic cell phenotype to the NSG line of mice that has been described as a maturation or functional defect (7). Many functional abnormalities have been described to monocytic cells in NOD mice, which have not been examined in NSG-MaFIA mice. However, the clearance of human red cells from the circulation of NSG-MaFIA mice, without prior homodimerizer treatment, had similar kinetics as in NSG mice, both of which were slower when compared with immunocompetent MaFIA mice with normal macrophages. Similar observations were previously made comparing human cell transfusion in NSG mice to other immunocompetent mouse strains (10). Culture-derived DCs and peritoneal macrophages from NOD mice were also reported to have diminished phagocytic ability, possibly associated with defects in annexin V and Fc-receptor function observed in NOD monocytic cells (26, 27).

We also observed a more rapid engraftment of human leukocytes in NSG-MaFIA mice that is consistent with past observations that phagocytes are responsible for the rapid depletion of human cells from the circulation of immunodeficient mice (11, 12, 28, 29). As macrophages and DCs are one of the barriers to engraftment of xenogeneic cells in NSG mice, it is possible that NSG-MaFIA mice may allow for improved engraftment of xenogeneic donor cells. Ongoing studies indicate that recovery of blood monocytes from homodimerizer treatment in NSG-MaFIA mice occurs within 2–3 weeks but that recovery of tissue macrophage and DC populations can take longer (work in progress). How this period of enhanced immunodeficiency can be utilized to promote engraftment of xenogeneic cells has not yet been studied.

EGFP expression was detected in myeloid cells with a clear gene-dosage effect observed. Interestingly, the intensity of EGFP expression in monocytes from MaFIA mice was modestly greater than in NSG-MaFIA mice. We suspect that the lower EGFP levels in NSG-MaFIA mice may result from the same maturation defect that affects many other aspects of monocytic cell function in mice on the NOD genetic background (7). Lower MaFIA transgene expression in the monocytic cells of NSG-MaFIA mice may also account for modestly lower efficiency of monocytic cell depletion in these mice compared with MaFIA mice. It should be noted that homodimerizer treatment of hemizygous NSG-MaFIA mice, which had half the expression of EGFP (based on MFI), led to the same weight loss as in homozygous mice, similar levels of monocyte depletion in the blood, and >60% macrophage depletion in tissues. Peritoneal macrophages in hemizygous mice, being at the site of injection and exposed to the highest levels of homodimerizer, were more effectively depleted than splenic and bone marrow macrophages. Whether intravenous delivery of homodimerizer affects cell depletion differently in the different tissues of hemizygous and homozygous mice has not yet been tested.

EGFP expression was also observed in granulocytes, which are not known to be responsive to macrophage colony-stimulating factor (M-CSF) during the final stages of development (30). EGFP expression was noted in the granulocyte population of MaFIA mice as well, with levels modestly lower than that of monocytic cells (14). Although some of the EGFP expression may carry over from a precursor population in which CSF-1R is expressed, it is now known that *Csf-1r* mRNA is expressed in neutrophils but not translated into CSF-1R (31, 32). Transgenic mice with *Csf-1r-*driven marker genes, besides MaFIA mice, have also shown transgene expression in neutrophils and eosinophils (31, 33).

Weight loss was observed with homodimerizer treatment as previously reported (14). It was previously indicated that most of this weight loss was associated with the loss of the monocytic cells as the homodimerizer drug treatment did not affect the weight of C57BL6/J mice. However, we observed a nearly 6% weight loss in NSG mice treated with homodimerizer compared with an average 24.6% in NSG-MaFIA mice (n = 26) observed across all experiments. The weight loss in NSG mice occurred rapidly with the onset of treatment and did not result from a steady decline as in NSG-MaFIA mice. Thus, some of the weight loss in NSG mice is attributed to toxicity from the dimerization agent. Toxicity of AP20187 on pluripotent stem cells has been noted *in vitro* (34). Furthermore, intraperitoneal administration of the homodimerizer has been described to induce peritoneal adhesions that may lead to gastrointestinal complications and develop into weight loss (16). There is no specific amount of weight loss that has been defined as a maximum acceptable level for experiments in mice (35, 36). Nonetheless, weight loss above 20% generally requires additional consideration and justification to ensure that humane guidelines are followed. We did observe deaths among NSG-MaFIA mice treated with homodimerizer but also transfused with PBMCs. It is currently not known how much weight loss contributed to the deaths of these animals and how much could be attributed to xenogeneic graft versus host disease. A 5-day course of homodimerizer treatment was used throughout this study (14), which we consider is a maximal dose, and future experimental protocols may consider reducing this regime.

NSG-MaFIA mice lack functional C5, hemolytic complement. Wetsel et al. reported a 2-bp TA deletion in C5-deficient mouse strains (20), which was confirmed to be the same mutation in NSG mice (37). NSG and NSG-MaFIA strains were aligned perfectly at the site of the mutation suggesting that they both contain the C5 mutation, whereas C57B6/J and MaFIA strains include the 2-bp TA in their nucleotide sequence suggesting that they have functional C5. Despite the lack of C5 function, xenogeneic cells can still be impacted by other complement proteins. Opsonization of human RBCs by mouse complement 3 protein, which can be inhibited by cobra venom factor, leads to binding of the RBCs to murine phagocytes (38, 39), thus accounting for at least one mechanism by which macrophage depletion leads to prolonged circulation of human RBCs in mice.

Despite depletion of monocytic cells in NSG-MaFIA mice, negligible numbers of hCD14^+^ cells were found to engraft after transfusion of human PBMC. Engrafted cells consisted mostly of T and B cells in line with previous findings (21, 22). Multilineage engraftment is more readily attained in mice engrafted with hematopoietic stem cells (4, 8, 9, 15, 40–42). Human monocyte and macrophage growth and survival in NSG-MaFIA mice is likely limited by species-restrictive activity of several key growth and survival factors. M-CSF is one such cytokine, with mouse M-CSF being inactive on human cells whereas human M-CSF can affect mouse cells (43). M-CSF is produced by many cell types (44), and transplanted human hematopoietic cells can serve as a source of human cytokines that may act in an autocrine, paracrine, or hormonal fashion. Consequently, higher levels of human chimerism create an environment more supportive of human cells. Monocytic cells are a source of M-CSF with its expression increasing, along with its receptor, colony-stimulating factor 1 receptor (CSF-1R), in response to various stimuli including other myelopoietic cytokines (45–48). IL-34, another CSF-1R ligand (49), acts on mostly different cell populations (e.g., Langerhans cells and microglia) than M-CSF through differential regulation of its expression (50, 51). Unlike M-CSF, however, murine IL-34 can stimulate human cells (52), but this is not expected to compensate for the lack of M-CSF cross-species activity.

The immunocompetent MaFIA mouse has proven useful as a macrophage depletion model for studying the role of monocytic cells in diverse tissues and disease states. NSG-MaFIA mice may further help in studying monocytic cell biology. Additionally, their severe immunodeficiency makes them excellent hosts for allogeneic or xenogeneic transplants. We have recently used MaFIA and NSG-MaFIA mice to delineate expression of lymphoid antigens on lymphoid and myeloid cells aided by EGFP expression under the *Csf1r* promotor provided by the MaFIA transgene (JQ Tran, EJ Du, G Rivera-Torruco, AJ Hui, K Luong, S House, C Tse, A Tam, RP Jackman, MO Muench, manuscript submitted). The possibility of using NSG-MaFIA mice to construct improved humanized mice will require optimizing depletion regimes to balance the benefits of monocytic cell depletion with maintaining the overall health of the host animal. Replacing some of the critical monocytic cell functions with human cells would be ideal but may also require generation of NSG-MaFIA mice expressing critical myelopoietic growth factors. Taken together, NSG-MaFIA mice represent a new platform to study monocytic cells and, with refinement, could advance xenogeneic mouse models.

## Data Availability

The raw data supporting the conclusions of this article will be made available by the authors, without undue reservation.
