# Supplementary material for: Development of an immunodeficient mouse that allows for conditional ablation of monocytic cells
Source: Front Immunol. 2025 Nov 4;16:1699385. doi: 10.3389/fimmu.2025.1699385 (PMC12623201; doi:10.3389/fimmu.2025.1699385)
Supplement: Supplementary file 1 [file DataSheet1.pdf]

Supplemental Table 1: CBC values for NSG-MaFIA, NSG, and MaFIA mice

NSG-MaFIA (n = 10)							
Parameter	Units	Mean	Median	SD	Max	Min	Range
Age	days	88.4	85	16.6	133	69	64
White blood cell (WBC)	10^3 cells/µL	3.27	3.24	0.66	4.14	2.17	1.97
Neutrophil	10^3 cells/µL	2.66	2.78	0.52	3.43	1.78	1.65
Lymphocyte	10^3 cells/µL	0.08	0.08	0.03	0.13	0.03	0.1
Monocyte	10^3 cells/µL	0.46	0.44	0.20	0.89	0.18	0.71
Eosinophil	10^3 cells/µL	0.05	0.04	0.02	0.11	0.02	0.09
Basophil	10^3 cells/µL	0.04	0.04	0.02	0.07	0	0.07
Percent Neutrophils	%	81.32	82.55	5.31	88.3	71.8	16.5
Percent Lymphocytes	%	2.34	2.1	0.69	3.9	1.5	2.4
Percent Monocytes	%	13.72	13.6	4.53	21.4	7.5	13.9
Percent Eosinophils	%	1.49	1.35	0.50	2.9	1	1.9
Percent Basophils	%	1.13	1.05	0.46	1.8	0.3	1.5
Red blood cell (RBC)	10^6 cells/µL	9.41	9.57	0.44	9.83	8.39	1.44
Hemoglobin (HGB)	g/dL	15.82	15.95	0.63	16.6	14.4	2.2
Hematocrit (HCT)	%	44.58	45.1	1.82	46.5	40.6	5.9
Mean cell volume (MCV)	fL	47.35	47.2	0.59	48.4	46.6	1.8
Mean cell hemoglobin (MCH)	pg	16.8	16.85	0.26	17.2	16.3	0.9
Mean cell hemoglobin concentration (MCHC)	g/dL	35.47	35.45	0.37	36.2	34.8	1.4
Red blood cell distribution width (RDW-CV)	%	13.06	13.1	0.25	13.4	12.7	0.7
Platelet (PLT)	10^3 cells/µL	1126.6	1115	61.66	1218	1028	190
Mean platelet volume (MPV)	fL	5.18	5.2	0.07	5.3	5	0.3

NSG (n = 10)							
Parameter	Units	Mean	Median	SD	Max	Min	Range
Age	days	59.6	64	10.0	66	40	26
White blood cell (WBC)	10^3 cells/µL	2.30	2.21	0.39	3.01	1.66	1.35
Neutrophil	10^3 cells/µL	1.86	1.83	0.32	2.48	1.35	1.13
Lymphocyte	10^3 cells/µL	0.03	0.03	0.03	0.09	0	0.09
Monocyte	10^3 cells/µL	0.34	0.36	0.10	0.51	0.16	0.35
Eosinophil	10^3 cells/µL	0.03	0.03	0.01	0.04	0.01	0.03
Basophil	10^3 cells/µL	0.04	0.04	0.02	0.08	0.01	0.07
Percent Neutrophils	%	80.56	80.9	2.87	86.8	76.6	10.2
Percent Lymphocytes	%	1.6	1.55	1.18	3.4	0	3.4
Percent Monocytes	%	14.49	14.45	2.95	18.1	7.6	10.5
Percent Eosinophils	%	1.4	1.3	0.46	2.2	0.8	1.4
Percent Basophils	%	1.95	1.75	1.06	4.3	0.5	3.8
Red blood cell (RBC)	10^6 cells/µL	9.66	9.67	0.49	10.45	8.63	1.82
Hemoglobin (HGB)	g/dL	16.4	16.45	0.70	17.7	15.2	2.5
Hematocrit (HCT)	%	46.81	46.95	1.94	50.5	43.4	7.1
Mean cell volume (MCV)	fL	48.48	48.2	0.76	50.2	47.5	2.7
Mean cell hemoglobin (MCH)	pg	17	16.9	0.28	17.6	16.7	0.9
Mean cell hemoglobin concentration (MCHC)	g/dL	35.08	35.15	0.17	35.3	34.7	0.6
Red blood cell distribution width (RDW-CV)	%	13.1	13	0.44	13.8	12.5	1.3
Platelet (PLT)	10^3 cells/µL	1237.5	1245	123.18	1461	1015	446
Mean platelet volume (MPV)	fL	5.17	5.2	0.13	5.3	4.9	0.4

MaFIA (n = 10)							
Parameter	Units	Mean	Median	SD	Max	Min	Range
Age	days	96.1	102	37.5	145	55	90
White blood cell (WBC)	10^3 cells/μL	11.91	13.26	2.92	14.96	7.37	7.59
Neutrophil	10^3 cells/μL	1.60	1.56	0.64	2.64	0.87	1.77
Lymphocyte	10^3 cells/μL	9.78	10.34	2.49	12.47	5.84	6.63
Monocyte	10^3 cells/μL	0.38	0.36	0.10	0.55	0.23	0.32
Eosinophil	10^3 cells/μL	0.10	0.10	0.04	0.18	0.04	0.14
Basophil	10^3 cells/μL	0.05	0.05	0.02	0.1	0.02	0.08
Percent Neutrophils	%	13.49	11.8	4.28	20.8	7.4	13.4
Percent Lymphocytes	%	82.01	83.35	4.46	88.9	74.4	14.5
Percent Monocytes	%	3.26	3.1	0.58	4.2	2.3	1.9
Percent Eosinophils	%	0.82	0.75	0.32	1.5	0.3	1.2
Percent Basophils	%	0.42	0.4	0.12	0.6	0.3	0.3
Red blood cell (RBC)	10^6 cells/μL	10.55	9.98	1.09	12.33	9.61	2.72
Hemoglobin (HGB)	g/dL	16.36	16.1	1.43	18.6	14.5	4.1
Hematocrit (HCT)	%	46.33	45.9	3.72	52.3	41	11.3
Mean cell volume (MCV)	fL	44.05	43.95	1.70	46.4	41.4	5
Mean cell hemoglobin (MCH)	pg	15.55	15.4	0.51	16.3	14.9	1.4
Mean cell hemoglobin concentration (MCHC)	g/dL	35.3	35.4	0.35	35.9	34.7	1.2
Red blood cell distribution width (RDW-CV)	%	12.42	12.35	0.61	13.9	11.8	2.1
Platelet (PLT)	10^3 cells/μL	1094.4	1010.5	219.63	1515	897	618
Mean platelet volume (MPV)	fL	5.37	5.35	0.17	5.6	5.1	0.5
